# Prevalence and prognostic implications of myocardial injury across different waves of COVID-19

**DOI:** 10.3389/fcvm.2024.1297824

**Published:** 2024-02-22

**Authors:** Óscar M. Peiró, Juan R. Delgado-Cornejo, Raúl Sánchez-Giménez, Víctor del-Moral-Ronda, Nisha Lal-Trehan, Mar Rocamora-Horrach, Anna Carrasquer, Joaquim Peraire, Isabel Fort-Gallifa, Alfredo Bardaji

**Affiliations:** ^1^ Department of Cardiology, Joan XXIII University Hospital, Tarragona, Spain; ^2^ Pere Virgili Health Research Institute, Rovira i Virgili University, Tarragona, Spain; ^3^ Department of Medicine and Surgery, Rovira i Virgili University, Tarragona, Spain; ^4^ Department of Internal Medicine, Joan XXIII University Hospital, Tarragona, Spain; ^5^ CIBER Enfermedades Infecciosas (CIBERINFEC)-Instituto de Salud Carlos III, Madrid, Spain; ^6^ Clinical Laboratory, Catalan Institute of Health, Camp de Tarragona-Terres de l’Ebre, Tarragona, Spain

**Keywords:** COVID-19, SARS-CoV-2, myocardial injury, troponin, prognostic

## Abstract

**Introduction:**

The prognostic ability of myocardial injury across different waves of the COVID-19 pandemic is not well established. The purpose of this study was to evaluate the prevalence and prognostic implications of myocardial injury in the first and sixth wave of COVID-19.

**Methods:**

We conducted a retrospective observational study that included patients admitted to the emergency department with COVID-19 with data on concentrations of cardiac troponin during the first and sixth wave. We compared the prevalence of myocardial injury and its predictive capacity for 30-day all-cause death in both waves.

**Results and discussion:**

A total of 346 patients were included (1st wave 199 and 6th wave 147 patients). The prevalence of myocardial injury was 21% with non-significant differences between waves. Myocardial injury was associated, in both waves, with a higher prevalence of comorbidities and with an increased risk of 30-day all-cause death [1st wave HR: 3.73 (1.84–7.55); *p* < 0.001 and 6th wave HR: 3.13 (1.23–7.92); *p* = 0.016], with non-significant differences in predictive capacity between groups after ROC curve analysis [AUC: 1st wave 0.829 (95% CI: 0.764–0.895) and 6th wave 0.794 (95% CI: 0.711–0.876)]. As limitations, this is a retrospective study with a relatively small simple size and troponin assay was performed at the discretion of the emergency physician so selection bias could be present. In conclusion, the prevalence of myocardial injury and its prognostic capacity was similar in both waves despite vaccination programs. Myocardial injury predicts short-term mortality in all COVID-19 patients, so they should be treated intensively.

## 1 Introduction

The coronavirus pandemic has become a global health emergency, causing extensive damage to many people's physical and mental well-being (1). As the virus spreads, it is crucial to understand its potential acute and long-term effects on the body (2). One of the most concerning effects is its potential to damage the heart muscle, known as myocardial injury (3). Much of the information about myocardial injury and COVID-19 comes from studies conducted during the first wave of infections in the first half of 2020. In this first wave, when the population had not been vaccinated or previously exposed to the virus, the health system became overloaded. During this period, the prevalence of myocardial injury was around 25% among COVID-19 patients. They showed higher prevalence of cardiovascular risk factors, severe course of the disease and higher risk of mortality (4, 5).

Around the world, we have had several waves of COVID-19 contagion (6). With each subsequent wave of contagion, the percentage of vaccinated population has increased (7), while new variants of the virus have appeared that are less aggressive (8). In addition, treatments that can mitigate the stormy evolution of the disease in high-risk patients have been developed (9). As a result, mortality in infected and hospitalized patients has decreased (10). In this context, it is necessary to reevaluate the analysis of myocardial injury in COVID-19 because the pandemic has been changing over time with an increase in the vaccinated population, new variants of the virus and new treatments.

At the end of 2021 and the beginning of 2022, the Spanish population suffered the well-known “sixth wave of contagion” that put the whole health system under pressure. It almost saturated the capacity of conventional hospital admissions and Intensive Care Units around the country. Despite high percentage of vaccination among Spanish population, the sixth wave was characterized by the highest incidence of COVID-19 in the entire pandemic. Given this scenario, we wonder if myocardial injury in patients with COVID-19 was similar to that detected in the first wave and, more importantly, if this myocardial injury continues to be a valuable predictor of mortality for risk stratification in patients attending emergency departments. To address these gaps in knowledge, our study aimed to compare the prevalence of myocardial injury and assess its prognostic implications for patients during the first and sixth waves of the COVID-19 pandemic.

## 2 Materials and methods

### 2.1 Study population

We conducted a retrospective observational study that included patients with definitive diagnosis of COVID-19 admitted to the emergency department of a tertiary hospital. The diagnosis of COVID-19 was confirmed if patients presented clinical signs and symptoms suggestive of COVID-19 and had a positive test for SARS-CoV-2 infection. During the first wave of COVID-19 we included patients from 16 March 2020 to 15 May 2020 and during the sixth wave from 1 December 2021 to 28 February 2022. These specific time periods correspond to the peak incidence of COVID-19 in our hospital for both waves. All patients had symptoms and a confirmed laboratory test of COVID-19 and, also, an available concentration of high-sensitivity cardiac troponin I (cTnI). If a patient had several cTnI determinations, the maximal value was selected. Patients with type 1 myocardial infarction were excluded because type 1 myocardial infarction reflects a different mechanism of myocardial injury that is not the predominant one among patients with COVID-19 and, also, because its clinical management is totally different (51 patients were excluded for type 1 myocardial infarction).

At the emergency department, patients were assessed for their clinical condition and risk factors. Clinical status and risk factors were evaluated by emergency physicians through a detailed anamnesis and physical examination, as well as an in-depth assessment of their electronic medical record. Patients with minor symptoms, good clinical status and no high-risk factors were discharged and followed up by their primary care physician, while those with moderate to severe clinical presentation were admitted to the Internal Medicine Department or Intensive Care Unit as appropriate. In first wave different treatments were administered (e.g., hydroxychloroquine, lopinavir/ritonavir, azithromycin, ACE inhibitors), however none of them demonstrated efficacy against COVID-19. During sixth wave, patients who were discharged due to good clinical status did not receive any specific treatment for COVID-19 and patients who were admitted to the Internal Medicine Department or Intensive Care Unit received dexamethasone and tocilizumab or remdesivir. Assessment of cTnI concentrations was performed at the discretion of the emergency physician; however, our hospital guidelines recommended cTnI measurement in all COVID-19 patients. Relevant clinical information were recorded on electronical medical records during the emergency department visit and hospital admission. Researchers thoroughly reviewed this information and compiled it into our database. The database was designed with several filters that avoid errors and, before carrying out the statistical study, an exhaustive analysis of the accuracy and completeness of the data was carried out. The primary endpoint was 30-day all-cause death, and there were no secondary endpoints. Deaths were identified by review of electronic medical records or telephone interview if electronic medical records were not available. Follow-up and death adjudications was performed by investigators who were blinded to cTnI measurements.

### 2.2 Laboratory analysis

To confirm SARS-CoV-2 infection polymerase chain reaction assays of nasal and pharyngeal swab specimens were performed. However, during the first wave antigen determination of nasal and pharyngeal swab specimens or plasma determination of antibodies were also performed.

Viral RNA purification was performed using the RNeasy Mini Kit in the Qiacube Connect (QIAGEN, Hilden, Germany). The reverse transcription polymerase chain re-action was performed with the thermocycler CFX96 Touch System (Bio-Rad Laboratories Inc., Hercules, CA) and a commercial kit intended to amplify regions of the E, N and RdRP genes (AllplexTM 2019-nCoV Assay, Seegene Inc., Seoul, South Korea). Antigen determination was performed by immunochromatography (Fluorescence Ag Rapid TestVR, BIOEASY Biotechnology Co., Ltd., Shenzhen, China), while antibodies were assessed by indirect chemiluminescent immunoassay (COVID-19 VIRCLIA Monotest, Vircell S.L., Granada, Spain).

cTnI concentrations were determined with an automated immunoassay (High-Sensitivity Troponin I Assay, Advia Centaur, Siemens Healthineers, Erlangen, Germany). As described by the manufacturer, the detection limit of the assay is 2.5 ng/L and the upper limit of detection is 25,000 ng/L (measured with a coefficient of variation <10%). Myocardial injury was defined as an elevated cTnI value above the 99th percentile of upper reference limit which corresponds to 47 ng/L.

### 2.3 Statistical analysis

Categorical variables are expressed as numbers and percentages, whereas continuous variables are expressed as medians and interquartile ranges (IQR). Comparisons of categorical data were performed with the *χ*^2^ test while numerical data was analysed with the Mann–Whitney *U-*test. Survival probabilities were estimated by the Kaplan–Meier method and compared with the log-rank test. To determine if cTnI was associated with 30-day all-cause death, univariable and multivariable Cox regressions were performed with the backward stepwise procedure. In multivariable analysis, clinically relevant and significant variables identified in the univariable analysis were included. Multivariable analysis included age, diabetes mellitus, chronic pulmonary disease, estimated glomerular filtration rate at admission and myocardial injury. The proportional hazards assumption was determined by the Schoenfeld residuals. Multicollinearity was examined by calculating the variance inflation factor. Finally, to assess the ability of cTnI to predict 30-day all-cause death, we performed ROC curve analyses. Statistically significant differences were considered if *p* < 0.05. STATA 14.2 (StataCorp, College Station, TX) was used for statistical analysis.

## 3 Results

### 3.1 Baseline characteristics

A total of 346 patients were included in the study, of which 199 (57.5%) were included during the first wave and 147 (42.5%) during the sixth wave. The median (IQR) age was 67.5 (54.5–80.5) years, and 153 (44.2%) patients were female. Baseline characteristics of patients from both waves were similar, but some differences need to be highlighted. Patients from the sixth wave were older and more frequently women, and had a higher prevalence of hypertension, hypercholesterolemia and medical history of heart failure (Table 1). During admission, patients in the sixth wave had a worse estimated glomerular filtration rate but patients in the first wave needed hospital admission, intensive care or mechanical ventilation more frequently (Table 2).

**Table 1:** Demographics, cardiovascular risk factors and medical history by COVID-19 waves.

Variable	Overall (*N* = 346)	1st wave (*N* = 199)	6th wave (*N* = 147)	*p*-value
Demographics				
Age, years	67.5 (54.5–80.5)	67.5 (53.5–78.5)	71.5 (56.5–84.5)	0.017
Female sex	153 (44.2)	79 (39.7)	74 (50.3)	0.049
Cardiovascular risk factors				
Current or past smoker	58 (16.8)	42 (21.1)	16 (10.9)	0.012
Hypertension	176 (50.9)	89 (44.7)	87 (59.2)	0.008
Diabetes mellitus	85 (24.6)	48 (24.1)	37 (25.2)	0.823
Hypercholesterolemia	108 (31.2)	51 (25.6)	57 (38.8)	0.009
Medical history				
Myocardial infarction	43 (12.4)	20 (10.1)	23 (15.7)	0.119
Heart failure	38 (11.0)	15 (7.5)	23 (15.7)	0.017
Cerebrovascular disease	27 (7.8)	14 (7.0)	13 (8.8)	0.535
Peripheral arterial disease	24 (7.0)	13 (6.5)	11 (7.5)	0.718
Chronic kidney disease	48 (13.9)	23 (11.6)	25 (17.0)	0.147
Chronic pulmonary disease	65 (18.8)	33 (16.6)	32 (21.8)	0.222
SARS-CoV-2 vaccination	112 (32.4)	0 (0.0)	112 (76.2)	<0.001

Data represent the number (percentage) or median (interquartile range).

**Table 2:** Clinical characteristics by COVID-19 waves.

Variable	Overall (*N* = 346)	1st wave *N* = 199)	6th wave (*N* = 147)	*p-*value
Symptoms				
Dyspnoea	203 (59.7)	117 (58.8)	86 (61.0)	0.684
Fever	206 (60.8)	142 (72.5)	64 (44.8)	<0.001
Cough	141 (41.8)	101 (51.5)	40 (28.4)	<0.001
Myalgias	50 (14.8)	11 (5.6)	39 (27.5)	<0.001
Diarrhoea	29 (8.8)	28 (14.3)	1 (0.8)	<0.001
Chest pain	30 (8.8)	17 (8.5)	13 (9.2)	0.828
Other symptoms	122 (36.0)	93 (46.7)	29 (20.7)	<0.001
Time from symptoms onset to admission (days)	4 (2–7)	4 (2–7)	3 (2–8)	0.840
Physical examination				
Systolic arterial pressure (mmHg)	120 (110–136)	124 (109–138)	120 (110–133)	0.332
Heart rate (bpm)	88 (76–103)	87 (74–104)	90 (78–102)	0.274
Oxygen saturation (%)	95 (92–98)	96 (92–99)	95 (92–97)	0.076
Electrocardiogram				
Atrial fibrillation	25 (7.2)	19 (9.6)	6 (4.1)	0.052
LBBB or RBBB	9 (2.6)	8 (4.0)	1 (0.7)	0.084
Radiology				
Consolidation	49 (14.6)	39 (19.6)	10 (7.3)	0.002
Ground-glass opacity	22 (6.6)	18 (9.1)	4 (2.9)	0.027
Bilateral pulmonary infiltration	220 (65.7)	124 (62.9)	96 (69.6)	0.209
Laboratory findings				
Glycemia (mg/dl)	110 (93–140)	105 (90–138)	112 (97–142)	0.051
eGFR (ml/min per 1.73 m^2^)	84 (54–101)	87 (58–103)	77 (46–96)	0.034
Renal impairment at admission	102 (29.5)	53 (26.6)	49 (33.3)	0.177
Haemoglobin (g/dl)	12.6 (11.2– 13.9)	12.6 (11.2– 13.9)	12.8 (11.2–13.8)	0.675
Leucocytes (×10^9^/L)	6.6 (4.9–8.8)	6.4 (4.7–9.0)	6.9 (5.3–8.4)	0.315
Lymphocytes (×10^9^/L)	0.9 (0.6–1.4)	0.8 (0.5–1.4)	1.0 (0.7–1.4)	0.038
Cardiac troponin *I* (ng/L)	14 (4–37)	14 (4–37)	13 (5–36)	0.997
Cardiac troponin *I* ≥ 47 ng/L	74 (21.4)	43 (21.6)	31 (21.1)	0.907
Clinical evolution				
Hospital admission	259 (75.7)	168 (84.4)	91 (63.6)	<0.001
ICU admission	49 (14.5)	36 (18.1)	13 (9.4)	0.025
Invasive mechanical ventilation	37 (11.0)	31 (15.6)	6 (4.4)	0.001
Mortality				
30-day all-cause death	59 (17.1)	38 (19.1)	21 (14.3)	0.240

Data represent the number (percentage) or median (interquartile range).

LBBB, indicates left bundle branch block; RBBB, right bundle branch block; eGFR, estimated glomerular filtration rate; ICU, intensive care unit.

The prevalence of SARS-CoV-2 vaccination was 76.2% (112 patients) in the sixth wave, while in the first wave no patients had been vaccinated. Of the 112 vaccinated patients, 52 had received three doses, 53 had received two doses and 7 had received one dose. Among patients of the sixth wave with myocardial injury there was a non-significant higher prevalence of vaccination against SARS-CoV-2 (Table 1 and Supplementary Table S1). Among patients that had received one dose; 4 patients had received Ad26.COV2.S (Janssen Johnson & Johnson), 2 patients BNT162b2 (Pfizer–BioNTech) and 1 patient mRNA-1273 (Moderna). Regarding patients who had received two doses; 14 patients had received ChAdOx1 nCoV-19 (AstraZeneca), 10 patients mRNA-1273 (Moderna) and 29 patients BNT162b2 (Pfizer–BioNTech). And as for patients who had received 3 doses; 34 patients had received BNT162b2 (Pfizer–BioNTech), 8 patients mRNA-1273 (Moderna), 9 patients a combination of two doses of BNT162b2 (Pfizer–BioNTech) and one dose of mRNA-1273 (Moderna) and 1 patient had received a combination of two doses of ChAdOx1 nCoV-19 (AstraZeneca) and one dose of mRNA-1273 (Moderna).

Regarding myocardial injury no differences were seen between the two waves. They showed a similar concentration of cTnI and prevalence of myocardial injury. However, patients of both waves with myocardial injury had a higher burden of cardiovascular risk factors and history of cardiovascular diseases than those patients without myocardial damage (Supplementary Table S1). Patients with myocardial injury also presented a worse estimated glomerular filtration rate at admission and worse clinical evolution during admission (Supplementary Table S2). Only one case of myocarditis and pulmonary thromboembolism was observed and both in the first wave.

### 3.2 30-day all-cause death endpoint

At 30 days of follow-up, 59 (17.1%) patients were dead: 38 (19.1%) died in the first wave and 21 (14.3%) in the sixth wave. In the first wave, there were 16 (10.3%) deaths among patients without myocardial injury and 22 (51.2%) deaths among patients with myocardial injury. During the sixth wave there were 10 (8.6%) deaths among patients without myocardial injury and 11 (35.5%) deaths among patients with myocardial injury. Mortality was more frequent during the first wave, especially in those with myocardial injury where significant difference were observed between waves (*p* < 0.001).

**Figure 1:**
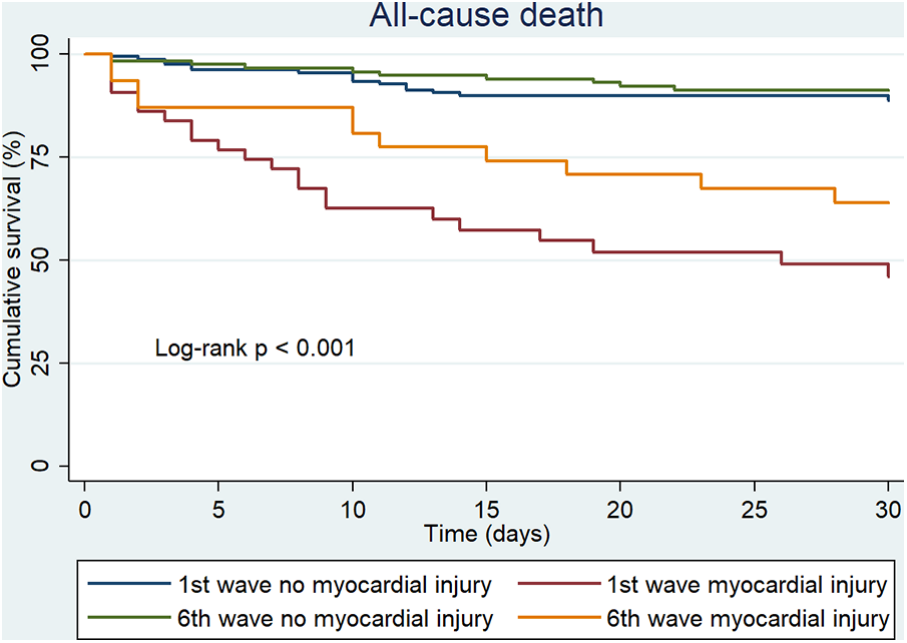
30-day all-cause death in 1st and 6th wave by presence of myocardial injury.

In the multivariable regression analysis the presence of myocardial injury was associated with 30-day all-cause mortality with a HR of 3.73 (95% CI: 1.84–7.55; *p* < 0.001) in the first wave and 3.13 (95% CI: 1.23–7.92; *p* = 0.016) in the sixth wave (Table 3 and Figure 1). The effect of vaccination, different types of vaccination and specific treatment for COVID-19 were explored in a multivariable analysis in the sixth wave group and no differences were found.

**Table 3:** Hazard ratios associated with all-cause death in univariate and multivariate Cox regression analysis by COVID-19 waves.

1st wave				
Variables	Univariate Cox Regression		Multivariate Cox Regression	
	HR (95% CI)	*p*-value	HR (95% CI)	*p*-value
Age	1.05 (1.03–1.07)	<0.001	–	–
Diabetes mellitus	2.06 (1.06–3.98)	0.032	–	–
Chronic pulmonary disease	2.94 (1.50–5.75)	0.002	2.56 (1.30–5.02)	0.007
eGFR at admission*	1.03 (1.02–1.04)	<0.001	1.02 (1.01–1.03)	<0.001
Myocardial injury	6.39 (3.35–12.19)	<0.001	3.73 (1.84–7.55)	<0.001
6th wave				
Variables	Univariate Cox Regression		Multivariate Cox Regression	
	HR (95% CI)	*p*-value	HR (95% CI)	*p*-value
Age	1.05 (1.02–1.08)	0.003	1.03 (1.00–1.07)	0.045
Diabetes mellitus	1.93 (0.80–4.65)	0.144	–	–
Chronic pulmonary disease	1.47 (0.57–3.78)	0.428	–	–
eGFR at admission*	1.02 (1.00–1.03)	0.020	–	–
Myocardial injury	4.77 (2.02–11.25)	<0.001	3.13 (1.23–7.92)	0.016

* Risk per point reduction in glomerular filtration rate.

HR, indicates hazard ratio; CI, confidence interval; eGFR, estimated glomerular filtration rate.

**Figure 2:**
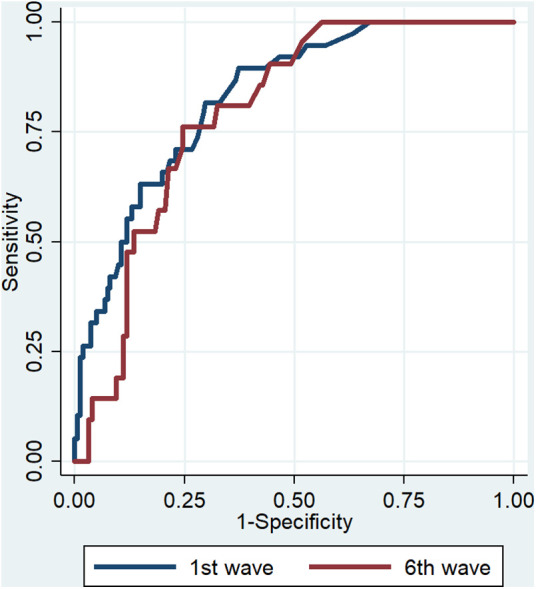
ROC curves for predicting 30-day all-cause death.

ROC curves were performed to determine if cTnI provided better prediction of 30-day all-cause death in the first or sixth wave. The AUC was 0.829 (95% CI: 0.764–0.895) in the first wave and 0.794 (95% CI: 0.711–0.876) in the sixth wave, with no significant difference between waves (*p* = 0.507; Figure 2).

## 4 Discussion

In this study we analysed the prevalence and prognostic implications of myocardial injury during the first and sixth waves of COVID-19. We found that myocardial injury was similar through waves of COVID-19 despite the high prevalence of vaccination in patients in the sixth wave. Moreover, we observed that patients of both waves exhibiting myocardial injury, had a higher prevalence of cardiovascular risk factors and history of cardiovascular diseases than those without myocardial injury. And, finally as main finding, we demonstrated that the presence of myocardial injury was associated with an increased risk of 30-day all-cause death being this association similar in both waves despite the adoption of massive vaccination. Therefore, our study provides new clinical evidence that confirm the important prognostic role of myocardial injury in patients with COVID-19.

Since the outbreak of the COVID-19 pandemic, healthcare systems around the world have been under extreme pressure. Due to the characteristics of the virus' spread and measures implemented to stop its progress, there have been different periods of time where the incidence of the virus has been higher, known as waves. Like other countries, in Spain there have been two waves of special interest: the first wave (beginning of pandemic to 21st June 2020) and the sixth wave (14th October 2021 to 27th March 2022). The first wave was especially important because COVID-19 was a new disease with no specific treatment, a high contagion rate and high mortality. The sixth wave was also very important because there was a very high incidence of infections despite the widespread uptake of vaccines and the availability of specific treatments (11, 12). Another reason why these two waves were important is because there was a major change in the main circulating variant of SARS-CoV-2. On 26 November 2021, the World Health Organization identified the new Omicron variant as a variant of concern that later became predominant worldwide, as was the case in Spain at the end of 2021 and beginning of 2022 (13). The Omicron variant presented multiple mutations of the spike protein that led to increased transmissibility and a decreased immune response after natural infection or vaccination (14, 15). For that reason, as the evolution of the COVID-19 pandemic and our preparation to fight the disease changes, it is important to assess whether the prognostic tools that were validated at the start of the pandemic are still working correctly.

At the beginning of the pandemic, there was medical concern about the degree of damage that the virus could cause to the heart. Although some advances have been made there are still gaps in knowledge that should be elucidated. Now we know that there are several mechanisms by which the virus can produce myocardial injury. Myocarditis, stress cardiomyopathy, pulmonary embolism or an acute coronary syndrome are reported mechanisms of myocardial injury. However, in the majority of COVID-19 cases, myocardial injury is driven by the systemic inflammation, sepsis and severe hypoxia that produce the illness (16, 17). In our study population, myocardial injury was mainly related to the systemic inflammation of COVID-19 in comorbid patients since cases of myocarditis and pulmonary thromboembolism were very infrequent. Although vaccination was associated with an increase in cases of myocarditis, in our sixth wave study population there were no cases (18).

In the first wave, the reported prevalence of myocardial injury was almost 20%, and patients who had myocardial injury were associated with an increased risk of death (19). In fact, even very small elevations of circulating cardiac troponin were associated with short-term mortality (20) and provided better prognostic capacities than other biomarkers (21). Interestingly, here we demonstrate that the prevalence of myocardial injury remains around 20% in the sixth wave, similar to that observed in the first wave, and more important, that myocardial injury continues to be associated with a higher risk of short-term mortality. Remarkably, these findings were found in patients that needed less hospitalization, intensive care unit admission, and mechanical ventilation and had a lower 30-day mortality com-pared with patients in the first wave.

Our results may be seen as unexpected due to the high rate of vaccination among patients of the sixth wave. However, there are some details that could explain the observed prevalence of myocardial injury. First, the initial vaccines that were developed against SARS-CoV-2 were specifically design for the original strain of SARS-CoV-2 and for the alpha variant that predominated at the beginning of the pandemic (22–24). However, with the subsequent variants and, especially, for the Omicron variant the effectiveness of vaccines decreased significantly (25). Thus, in our study, sixth wave patients might not have been as protected as expected following the emergence of the Omicron variant. Secondly, it is not known the exact degree of injury that the virus produces in the myocardium with the subsequent variants of SARS-CoV-2. The Omicron variant has been associated to a less aggressive variant than the original strain or alpha variant. However, the Omicron variant might have lost aggressiveness for other organs such as the lungs but maintained aggressiveness to myocardial tissue. Therefore, a relatively more benign variant may not be as benign for the heart. And thirdly, sixth wave patients were older and had more comorbidities (hypertension, hypercholesterolemia, heart failure and worse estimated glomerular filtration rate) than first wave patients. So, myocardial injury may not only be a reflection of the acute pathology but could also be an indication of the patient's comorbidity burden (26). Altogether this could explain why the prevalence of myocardial injury was high in sixth wave patients. What is not unexpected is that myocardial injury continues to be a good prognostic biomarker that could help clinicians to identify those patients at higher risk of mortality. In fact, myocardial injury has proven to be an excellent stratification tool in several diseases (27) and, as demonstrated herein, it continues to be so in COVID-19.

There were some limitations in our study. It was a retrospective observational study developed in a single centre with patients that were admitted to an emergency department of a tertiary hospital. Although data collection was meticulously performed, minimal residual biases may exist due to the nature of a retrospective observational study. The sample size was relatively small. The cTnI assay was performed at the discretion of the emergency physician so selection bias could be present. Viral presence confirmation was detected mainly by polymerase chain reaction, however in 16% of patients from the first wave, viral presence was detected by antigens from nasal and pharyngeal swab samples or determination of antibodies in plasma. In a pandemic situation with high spread of the virus, a positive test for determination of antigens or antibodies in patients with compatible symptoms made a diagnostic error highly unlikely, yet false positive could be present. Although we know, from epidemiological studies, the predominant SARS-CoV-2 variant in our territory we are unaware of the exact prevalence of different variants in our population. The number of previous SARS-CoV-2 infections was not studied in sixth wave patients, and we do not know if the number of reinfections could affect our results. And finally, treatments for COVID-19 differed between the first wave and sixth wave, especially as unproven treatments were administered in the first wave, and we do not know if those treatments may have influenced our observed results.

## 5 Conclusions

Our study shows that the prevalence of myocardial injury during the first and sixth waves of COVID-19 was similar despite the implantation of vaccination of patients in the sixth wave. Furthermore, we demonstrated that the presence of myocardial injury was associated with an increased risk of 30-day all-cause death and that it provided a similar capacity for risk prediction in both waves. Therefore, myocardial injury continues to play an important role in risk stratification in COVID-19.

## Data Availability

The raw data supporting the conclusions of this article will be made available by the authors, without undue reservation.

## References

[1] Miyah Y Benjelloun M Lairini S Lahrichi A. COVID-19 impact on public health, environment, human psychology, global socioeconomy, and education. Sci World J. (2022) 2022:5578284. 10.1155/2022/5578284 PMC8767375 35069037

[2] Joshee S Vatti N Chang C. Long-term effects of COVID-19. Mayo Clin Proc. (2022) 97:579–99. 10.1016/j.mayocp.2021.12.017 35246288 PMC8752286

[3] Bardaji A. Myocardial injuries in COVID-19: more questions than answers. J Clin Med. (2022) 11(15):4527. 10.3390/jcm11154527 35956141 PMC9369937

[4] Bardají A Carrasquer A Sánchez-Giménez R Lal-Trehan N del-Moral-Ronda V Peiró ÓM Prognostic implications of myocardial injury in patients with and without COVID-19 infection treated in a university hospital. Revista Española de Cardiología (English Edition). (2021) 74:24–32. 10.1016/j.rec.2020.08.027 PMC7561309 33144126

[5] Li S Wang J Yan Y Zhang Z Gong W Nie S. Clinical characterization and possible pathological mechanism of acute myocardial injury in COVID-19. Front Cardiovasc Med. (2022) 9:862571. 10.3389/fcvm.2022.862571 35387441 PMC8979292

[6] Ratcliffe NA Castro HC Gonzalez MS Mello CB Dyson P. Reaching the final endgame for constant waves of COVID-19. Viruses. (2022) 14(12):2637. 10.3390/v14122637 36560641 PMC9783511

[7] Marco JJG Pasquín MJÁ. COVID-19 vaccination in Spain: successes, mistakes and future prospects. Aten Primaria. (2021) 53(10):102193. 10.1016/j.aprim.2021.102193 34678679 PMC8437804

[8] Zhang Y Zhang H Zhang W. SARS-CoV-2 variants, immune escape, and countermeasures. Front Med. (2022) 16:196–207. 10.1007/s11684-021-0906-x 35253097 PMC8898658

[9] Chavda VP Kapadia C Soni S Prajapati R Chauhan SC Yallapu MM A global picture: therapeutic perspectives for COVID-19. Immunotherapy. (2022) 14:351–71. 10.2217/imt-2021-0168 35187954 PMC8884157

[10] Khan ZS Van Bussel F Hussain F. Modeling the change in European and US COVID-19 death rates. PLoS One. (2022) 17(8):e0268332. 10.1371/journal.pone.0268332 35976910 PMC9385065

[11] Red Nacional de Vigilancia Epidemiológica. Centro Nacional de Epidemiología. COVID-19 situation in Spain. 117th report at February 9, 2022. (2022). Available at: https://www.isciii.es/QueHacemos/Servicios/VigilanciaSaludPublicaRENAVE/EnfermedadesTransmisibles/Documents/INFORMES/Informes%20COVID-19/INFORMES%20COVID-19%202022/Informe%20n%C2%BA%20117%20Situaci%C3%B3n%20de%20COVID-19%20en%20Espa%C3%B1a%20a%2009%20de%20febrero%20de%202022.pdf (accessed January 14, 2023).

[12] Red Nacional de Vigilancia Epidemiológica. Centro Nacional de Epidemiología. COVID-19 situation in Spain. 160th report at December 23, 2022. (2022). Available at: https://www.isciii.es/QueHacemos/Servicios/VigilanciaSaludPublicaRENAVE/EnfermedadesTransmisibles/Documents/INFORMES/Informes%20COVID-19/INFORMES%20COVID-19%202022/Informe%20n%C2%BA%20160%20Situaci%C3%B3n%20actual%20de%20COVID-19%20en%20Espa%C3%B1a%20a%2023%20de%20diciembre%20de%202022.pdf (accessed January 14, 2023).

[13] Centro de Coordinación de Alertas y Emergencias Sanitarias. SARS-CoV-2 variants in Spain. 9th update at January 18, 2022. (2022). Available at: https://www.sanidad.gob.es/profesionales/saludPublica/ccayes/alertasActual/nCov/documentos/20220118-ERR.pdf (accessed January 14, 2023).

[14] Zhou D Dejnirattisai W Supasa P Liu C Mentzer AJ Ginn HM Evidence of escape of SARS-CoV-2 variant B.1.351 from natural and vaccine-induced sera. Cell. (2021) 184:2348–61.e6. 10.1016/j.cell.2021.02.037 33730597 PMC7901269

[15] Dejnirattisai W Huo J Zhou D Zahradník J Supasa P Liu C SARS-CoV-2 omicron-B.1.1.529 leads to widespread escape from neutralizing antibody responses. Cell. (2022) 185:467–84.e15. 10.1016/j.cell.2021.12.046 35081335 PMC8723827

[16] Imazio M Klingel K Kindermann I Brucato A De Rosa FG Adler Y COVID-19 pandemic and troponin: indirect myocardial injury, myocardial inflammation or myocarditis? Heart. (2020) 106:1127–31. 10.1136/heartjnl-2020-317186 32499236

[17] Nishiga M Wang DW Han Y Lewis DB Wu JC. COVID-19 and cardiovascular disease: from basic mechanisms to clinical perspectives. Nat Rev Cardiol. (2020) 17(9):543–58. 10.1038/s41569-020-0413-9 32690910 PMC7370876

[18] Karlstad Ø Hovi P Husby A Härkänen T Selmer RM Pihlström N SARS-CoV-2 vaccination and myocarditis in a nordic cohort study of 23 million residents. JAMA Cardiol. (2022) 7(6):600–12. 10.1001/jamacardio.2022.0583 35442390 PMC9021987

[19] Shi S Qin M Shen B Cai Y Liu T Yang F Association of cardiac injury with mortality in hospitalized patients with COVID-19 in Wuhan, China. JAMA Cardiol. (2020) 5:802–10. 10.1001/jamacardio.2020.0950 32211816 PMC7097841

[20] Shi S Shi S Shi S Qin M Cai Y Liu T Characteristics and clinical significance of myocardial injury in patients with severe coronavirus disease 2019. Eur Heart J. (2020) 41:2070–9. 10.1093/eurheartj/ehaa408 32391877 PMC7239100

[21] Peiró ÓM Carrasquer A Sánchez-Gimenez R Lal-Trehan N del-Moral-Ronda V Bonet G Biomarkers and short-term prognosis in COVID-19. Biomarkers. (2021) 26:119–26. 10.1080/1354750X.2021.1874052 33426934 PMC7832452

[22] Polack FP Thomas SJ Kitchin N Absalon J Gurtman A Lockhart S Safety and efficacy of the BNT162b2 mRNA COVID-19 vaccine. N Engl J Med. (2020) 383:2603–15. 10.1056/nejmoa2034577 33301246 PMC7745181

[23] Baden LR El Sahly HM Essink B Kotloff K Frey S Novak R Efficacy and safety of the mRNA-1273 SARS-CoV-2 vaccine. N Engl J Med. (2021) 384:403–16. 10.1056/nejmoa2035389 33378609 PMC7787219

[24] Voysey M Clemens SAC Madhi SA Weckx LY Folegatti PM Aley PK Safety and efficacy of the ChAdOx1 nCoV-19 vaccine (AZD1222) against SARS-CoV-2: an interim analysis of four randomised controlled trials in Brazil, South Africa, and the UK. Lancet. (2021) 397:99–111. 10.1016/S0140-6736(20)32661-1 33306989 PMC7723445

[25] Andrews N Stowe J Kirsebom F Toffa S Rickeard T Gallagher E COVID-19 vaccine effectiveness against the omicron (B.1.1.529) variant. N Engl J Med. (2022) 386:1532–46. 10.1056/nejmoa2119451 35249272 PMC8908811

[26] Metkus TS Sokoll LJ Barth AS Czarny MJ Hays AG Lowenstein CJ Myocardial injury in severe COVID-19 compared with non-COVID-19 acute respiratory distress syndrome. Circulation. (2021) 143:553–65. 10.1161/CIRCULATIONAHA.120.050543 33186055 PMC7864609

[27] Bardají A Cediel G Carrasquer A De Castro R Sánchez R Carmen B. Troponin elevation in patients without acute coronary syndrome. Rev Esp Cardiol (Engl Ed). (2015) 68:469–76. 10.1016/j.recesp.2014.10.018 25800165

