# Supplementary material for: Prevalence and prognostic implications of myocardial injury across different waves of COVID-19
Source: Front Cardiovasc Med. 2024 Feb 22;11:1297824. doi: 10.3389/fcvm.2024.1297824 (PMC10917998; doi:10.3389/fcvm.2024.1297824)
Supplement: Supplementary file 1 [file Table1.docx]

Supplementary Material

# Supplementary tables

**Supplementary table 1. Demographics, cardiovascular risk factors and medical history by COVID-19 waves and myocardial injury.**

Data represent the number (percentage) or median (interquartile range).

|  | **1^st^ COVID-19 wave** | | **6^th^ COVID-19 wave** | | **P value** |
| --- | --- | --- | --- | --- | --- |
| **Variable** | **No myocardial injury**  **(N= 156)** | **Myocardial injury**  **(N = 43)** | **No myocardial injury**  **(N= 116)** | **Myocardial injury**  **(N = 31)** |  |
| **Demographics** | | | | | |
| Age, years | 61.5 (51.0-75.5) | 75.5 (64.5-86.5) | 66.5 (54.5-80.5) | 85.5 (73.5-91.5) | <0.001 |
| Female sex | 65 (41.7) | 14 (32.6) | 64 (55.2) | 10 (32.3) | 0.017 |
| **Cardiovascular risk factors** | | | | | |
| Current or past smoker | 28 (18.0) | 14 (32.6) | 14 (12.1) | 2 (6.5) | 0.007 |
| Hypertension | 58 (37.2) | 31 (72.1) | 60 (51.7) | 27 (87.1) | <0.001 |
| Diabetes mellitus | 31 (19.9) | 17 (39.5) | 26 (22.4) | 11 (35.5) | 0.025 |
| Hypercholesterolemia | 32 (20.5) | 19 (44.2) | 40 (34.5) | 17 (54.8) | <0.001 |
| **Medical history** | | | | | |
| Myocardial infarction | 7 (4.5) | 13 (30.2) | 13 (11.2) | 10 (32.3) | <0.001 |
| Heart failure | 8 (5.1) | 7 (16.3) | 15 (12.9) | 8 (25.8) | 0.003 |
| Cerebrovascular disease | 9 (5.8) | 5 (11.6) | 6 (5.2) | 7 (22.6) | 0.006 |
| Peripheral arterial disease | 5 (3.2) | 8 (18.6) | 5 (4.4) | 6 (19.4) | <0.001 |
| Chronic kidney disease | 8 (5.1) | 15 (34.9) | 16 (13.8) | 9 (29.0) | <0.001 |
| Chronic pulmonary disease | 26 (16.7) | 7 (16.3) | 23 (19.8) | 9 (29.0) | 0.415 |
| SARS-CoV-2 vaccination | 0 (0.0) | 0 (0.0) | 85 (73.3) | 27 (87.1) | <0.001 |

**Supplementary table 2. Clinical characteristics by COVID-19 waves and myocardial injury.**

|  | **1st COVID-19 wave** | | **6th COVID-19 wave** | | **P value** |
| --- | --- | --- | --- | --- | --- |
| **Variable** | **No myocardial injury**  **(N= 156)** | **Myocardial injury**  **(N = 43)** | **No myocardial injury**  **(N= 116)** | **Myocardial injury**  **(N = 31)** |  |
| **Symptoms** | | | | |  |
| Dyspnoea | 90 (57.7) | 27 (62.8) | 62 (55.4) | 24 (82.8) | 0.052 |
| Fever | 114 (74.0) | 28 (66.7) | 53 (47.3) | 11 (35.5) | <0.001 |
| Cough | 87 (56.5) | 14 (33.3) | 32 (28.8) | 8 (26.7) | <0.001 |
| Myalgias | 8 (5.2) | 3 (7.1) | 34 (30.4) | 5 (16.7) | <0.001 |
| Diarrhoea | 24 (15.6) | 4 (9.5) | 1 (1.0) | 0 (0.0) | <0.001 |
| Chest pain | 16 (10.3) | 1 (2.3) | 11 (9.8) | 2 (6.9) | 0.403 |
| Other symptoms | 76 (48.7) | 17 (39.5) | 27 (24.3) | 2 (6.9) | <0.001 |
| Time from symptoms onset to admission (days) | 5 (2 – 7) | 3 (0 – 7) | 4 (2 – 9) | 3 (1 – 4) | 0.025 |
| **Physical examination** | | | | |  |
| Systolic arterial pressure (mmHg) | 124 (111 - 135) | 128 (109 - 141) | 120 (112 - 133) | 120 (109 - 139) | 0.738 |
| Heart rate (bpm) | 88 (75 - 105) | 86 (74 - 99) | 91 (78 - 103) | 88 (77 – 100) | 0.554 |
| Oxygen saturation (%) | 96 (93 - 99) | 94 (85 - 98) | 95 (92 - 98) | 93 (90 – 95) | <0.001 |
| **Electrocardiogram** | | | | |  |
| Atrial fibrillation | 11 (7.1) | 8 (18.6) | 4 (3.5) | 2 (6.5) | 0.013 |
| LBBB or RBBB | 4 (2.6) | 4 (9.3) | 0 (0.0) | 1 (3.2) | 0.013 |
| **Radiology** | | | | |  |
| Consolidation | 31 (19.9) | 8 (18.6) | 6 (5.6) | 4 (13.3) | 0.012 |
| Ground-glass opacity | 14 (9.0) | 4 (9.3) | 2 (1.9) | 2 (6.7) | 0.123 |
| Bilateral pulmonary infiltration | 93 (60.0) | 31 (73.8) | 71 (65.7) | 25 (83.3) | 0.055 |
| **Laboratory findings** | | | | |  |
| Glycemia (mg/dL) | 102 (90 - 124) | 135 (96 - 157) | 112 (97 - 133) | 119 (93 - 171) | 0.002 |
| eGFR (mL/min per 1.73 m^2^) | 91 (73 - 104) | 48 (19 - 84) | 82 (62 - 100) | 37 (30 - 69) | <0.001 |
| Renal impairment at admission | 26 (16.7) | 27 (62.8) | 27 (23.3) | 22 (71.0) | <0.001 |
| Haemoglobin (g/dL) | 12.7 (11.7– 14.0) | 11.2 (9.5– 12.9) | 12.8 (11.6– 13.9) | 12.2 (10.1– 13.2) | 0.002 |
| Leucocytes (x 10^9^/L) | 6.0 (4.5 – 8.3) | 7.9 (5.9 – 10.7) | 6.6 (4.9 – 8.1) | 8.2 (6.8 – 10.8) | 0.001 |
| Lymphocytes (x 10^9^/L) | 0.9 (0.5 - 1.4) | 0.7 (0.4 - 1.2) | 1.0 (0.7 - 1.4) | 0.9 (0.6 - 1.2) | 0.806 |
| Cardiac troponin I (ng/L) | 9 (3 – 20) | 120 (69 – 368) | 9 (2 – 21) | 156 (73 – 647) | <0.001 |
| **Clinical evolution** | | | | |  |
| Hospital admission | 126 (80.8) | 42 (97.7) | 65 (58.0) | 26 (83.9) | <0.001 |
| ICU admission | 28 (18.0) | 8 (18.6) | 8 (7.3) | 5 (17.2) | 0.075 |
| Invasive mechanical ventilation | 22 (14.1) | 9 (20.9) | 3 (2.8) | 3 (10.0) | 0.004 |
| **Mortality** | | | | |  |
| 30-days death | 16 (10.3) | 22 (51.2) | 10 (8.6) | 11 (35.5) | <0.001 |

Data represent the number (percentage) or median (interquartile range). LBBB indicates left bundle branch block. RBBB: right bundle branch block; eGFR: estimated glomerular filtration rate; ICU: intensive care unit.
